# Burden of disease, treatment utilization, and the impact on education and employment in patients with sickle cell disease: A comparative analysis of high‐ and low‐ to middle‐income countries for the international Sickle Cell World Assessment Survey

**DOI:** 10.1002/ajh.26576

**Published:** 2022-06-20

**Authors:** Ifeyinwa Osunkwo, John James, Fuad El‐Rassi, Alecia Nero, Caterina P. Minniti, Cassandra Trimnell, Jincy Paulose, Nicholas Ramscar, Tom Bailey, Olivera Rajkovic‐Hooley, Biree Andemariam

**Affiliations:** ^1^ Sickle Cell Disease Enterprise, The Levine Cancer Institute, Atrium Health Charlotte North Carolina USA; ^2^ Sickle Cell Society London UK; ^3^ Emory University School of Medicine and Georgia Comprehensive Sickle Cell Center at Grady Health System Atlanta Georgia USA; ^4^ University of Texas Southwestern Medical Center Dallas Texas USA; ^5^ Albert Einstein College of Medicine New York New York USA; ^6^ Sickle Cell 101 San Jose California USA; ^7^ Novartis Pharmaceuticals Corporation East Hanover New Jersey USA; ^8^ Novartis Pharma AG Basel Switzerland; ^9^ Adelphi Real World Bollington UK; ^10^ New England Sickle Cell Institute University of Connecticut Health Farmington Connecticut USA; ^11^ Present address: Forma Therapeutics Watertown Massachusetts USA

## Abstract

The international Sickle Cell World Assessment Survey (SWAY) reported a high impact of sickle cell disease (SCD) on patients' daily lives globally. In this study, we analyzed whether the reported burden differed between patients from the USA (*n* = 384) and other high‐income (HI; *n* = 820) or low‐ to middle‐income (LMI; *n* = 941) countries. We assessed symptoms and complications, incidence/management of vaso‐occlusive crises (VOCs), treatment utilization/satisfaction, and the impact of SCD on education/employment. Certain symptoms (bone aches, insomnia, and joint stiffness) and complications (swollen/painful fingers/toes, gallstones, vision problems, blood clots, and asthma) were reported proportionally more by patients in the USA than in the HI/LMI countries. Self‐reported VOCs were more common (mean [SD]: 7.1 [5.7] vs. 5.5 [8.9] and 4.4 [4.6] in the previous 12 months) and were managed more often by hospitalization (52% vs. 24% and 32%) in the USA than the HI and LMI countries. A higher proportion of patients from the USA than the HI/LMI countries reported a negative impact of SCD on their employment/schooling. Although high overall satisfaction with current treatments was reported globally, most patients indicated a strong desire for alternative pain medications. There are likely several reasons for the relatively high patient‐reported burden in the USA group compared with the HI/LMI countries, including an older population and differences in newborn screening programs and pediatric/adult transition of care. It is clear that there is an urgent need for improved understanding and management of SCD globally, not just in the USA.

## 1 INTRODUCTION

Sickle cell disease (SCD) is an inherited blood disorder with a complex pathophysiology largely driven by vaso‐occlusion and hemolytic anemia.^1^ Patients with SCD may experience a range of symptoms and complications, including acute chest syndrome, infections, pulmonary hypertension, stroke, and painful vaso‐occlusive crises (VOCs).^2^ VOCs are the hallmark of SCD and the leading cause of emergency department (ED) visits and hospitalization,^2^ and are associated with a significantly elevated risk of life‐threatening organ damage and death.^3^, ^4^

Newborn screening programs for SCD are standard practice in many high‐income (HI) countries, including the USA, but are limited or non‐existent in many low‐ to middle‐income (LMI) countries. Therefore, the exact global prevalence of SCD is unknown.^3^ However, approximately, 300 000 infants are born each year with homozygous SCD (HbSS), with an estimated two‐thirds of these being in Africa.^5^ There is substantial disparity in life expectancy between HI (e.g., 54 years in the USA) and LMI countries (e.g., estimated at <20 years across Africa).^6^, ^7^ This is largely due to differential access to newborn screening programs and disparities in accessibility and availability of healthcare resources and treatments (particularly prophylactic antibiotics and hydroxyurea [HU]).

The international Sickle Cell World Assessment Survey (SWAY) was a cross‐sectional questionnaire that aimed to better inform the management and treatment of SCD, and support global healthcare policy and delivery, by collecting real‐world insights into the impact of SCD on patients' daily lives and the treatment they receive.^8^ The high impact of SCD on patients' lives previously reported by the SWAY investigators^8^ is consistent with multiple other studies,^9^, ^10^, ^11^, ^12^, ^13^ although these studies focus on restricted populations, including single countries,^9^, ^10^, ^11^, ^12^ single regions/centers,^9^, ^12^ and/or specific age groups.^13^ In contrast, SWAY included patients from different countries, socioeconomic backgrounds, ages, and genotypes.

The primary analysis of SWAY demonstrated that the unmet needs in SCD care and management differ substantially between HI and LMI countries.^8^ The aim of the current analysis was to compare data obtained from the USA with that from other HI and LMI countries, highlighting any differences in the reported disease burden, treatment utilization, and impact of SCD on education and employment.

## 2 METHODS

### 2.1 SWAY

Full methodological details have been described previously.^8^

#### 2.1.1 Survey design and objectives

Briefly, SWAY was a multi‐country, cross‐sectional survey conducted in 16 countries (Figure S1) between April 3 and October 4, 2019. The survey was developed by a global steering committee comprising 14 SCD expert physicians and three patient advocacy group (PAG) stakeholders, with input from Novartis representatives.

#### 2.1.2 Participants

Patients aged ≥6 years with a diagnosis of SCD participated in SWAY. Patients were recruited either by treating HCPs during routine consultations or via invitation from PAGs. A proxy (parent/guardian/caregiver) completed the survey for patients aged 6–11 years. Patients aged ≥12 years could complete the survey independently or seek input from a parent/guardian/caregiver, or a proxy could complete the survey for them. HCPs were also invited to take a separate survey, but only results from the patient survey are presented here.

#### 2.1.3 Survey questions

Collected data included: patient demographics, SCD symptoms (disease features resulting directly from SCD; excluding VOCs, which were assessed separately), complications (disease features that are a secondary result of SCD and that often require additional specialist care), incidence and management of VOCs, medications received for SCD (currently and ever), and satisfaction with these medications. The survey was completed prior to the approval of crizanlizumab (approved by the US Food and Drug Administration [FDA] in 2019 and European Medicines Agency in 2020) and voxelotor (approved by the FDA in 2019); therefore, these treatments were not included in the survey. Question format was predominantly multiple choice or ratings based. For ratings‐based questions, participants recorded their agreement with a given statement using a 7‐point Likert scale. The scale was defined for each question (e.g., 1 = “not at all,” 7 = “a great deal”; 1 = “not severe,” 7 = “worst imaginable”; 1 = “very dissatisfied,” 7 = “very satisfied”; or 1 = “strongly disagree,” 7 = “strongly agree”). A score of 5, 6, or 7 was deemed to indicate “high impact,” “high severity,” “high satisfaction,” or “strong agreement,” respectively.

### 2.2 Data analysis

All reported data and associated analyses are descriptive. All data comparisons are between the USA and other countries participating in SWAY, stratified according to economic status (HI or LMI). The World Bank definition for an HI economy (gross national income per capita of ≥US$12 536)^14^ was used to stratify countries. An arbitrary threshold of ≥10% is used to define differences in data between the USA and HI/LMI groups; any differences <10% are considered comparable.

## 3 RESULTS

### 3.1 Demographic data

A total of 2145 patients participated in SWAY, comprising 384 (18%) from the USA, 820 (38%) from other HI countries (Bahrain, Canada, France, Germany, Italy, the Netherlands, Oman, Panama, Saudi Arabia, and the UK), and 941 (44%) from LMI countries (Brazil, Ghana, India, Nigeria, and Lebanon) (Figure S1, Table 1).

**TABLE 1:** Demographic data for patients in SWAY

	USA (*n* = 384)	HI (*n* = 820)	LMI (*n* = 941)
Mean age, years	30	28	20^a^
Female, %	61	54	47
≤18 years, %	21	27	56
Mean age, years	12	13	12
Self‐reported genotype, %			
HbSS	53	53	43
HbSC	26	16	23
Hbβ^0^	5	3	1
Hbβ^+^	5	4	1
Other	1	1	2
Unknown	10	23	30
Range across group, %		3–71 (10 countries)	5–53 (5 countries)
Recruited via, %			
HCPs using ARW network	39	50	72
PAGs	61	50	28
Completed by proxy, %	20	25	43

Abbreviations: ARW, Adelphi Real World; HCP, healthcare professional; HI, high‐income; LMI, low‐ to middle‐income; PAG, patient advocacy group; SWAY, Sickle Cell World Assessment Survey.

^a^ *n* = 940; one person from Lebanon did not report their age.

Demographics and baseline characteristics were generally similar between the USA and HI groups, with more apparent differences between the USA and LMI groups (Table 1). Importantly, the mean age of the LMI group (20 years) was lower than that of the USA group (30 years). Additionally, we observed a higher proportion of participants who completed the survey by proxy in the LMI group (43%) than in the USA group (20%). Interestingly, fewer patients in the USA group (10%) self‐reported their genotype as unknown than in the HI (23%; range across 10 countries: 3%–71%) and LMI (30%; range across five countries: 5%–53%) groups; this may reflect the lack of consistency in the availability of SCD screening programs globally.

### 3.2 Symptoms and complications (excluding VOCs)

**FIGURE 1:**
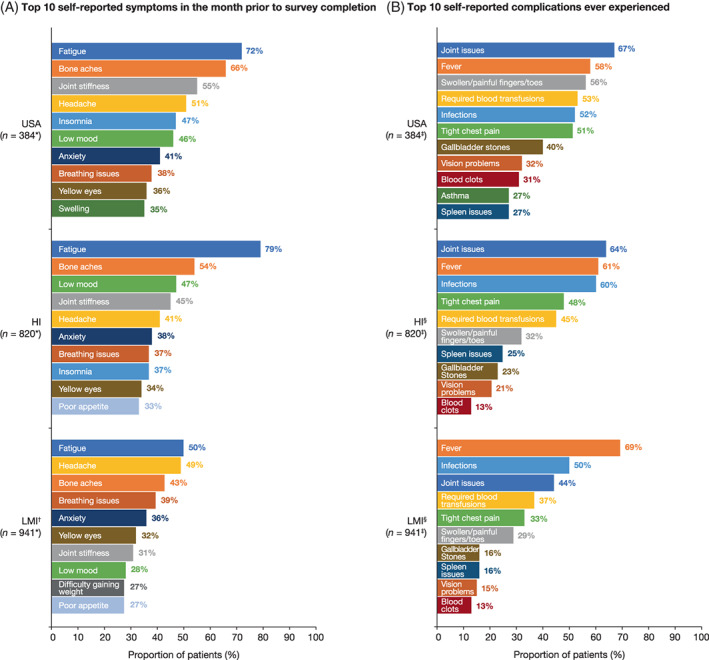
Top 10 self‐reported (A) symptoms experienced in the month prior to survey completion and (B) complications ever experienced by patients with sickle cell disease in the USA, HI, and LMI groups. *This included patients who selected ‘No symptoms’ (USA, *n* = 3; HI, *n* = 21; and LMI, *n* = 29). ^†^Insomnia, reported by 26% of patients, was outside of the top 10 symptoms for the LMI group. ^‡^This included patients who selected ‘No complications’ (USA, *n* = 3; HI, *n* = 40; and LMI, *n* = 62). ^§^Asthma, reported by 11% and 8% of patients, was outside of the top 10 complications for the HI and LMI groups, respectively. HI, high‐income; LMI, low‐ to middle‐income [Color figure can be viewed at wileyonlinelibrary.com]

Self‐reported symptoms experienced in the month prior to survey completion were generally consistent across groups. Anxiety, bone aches, breathing issues, fatigue, joint stiffness, headache, low mood, and yellow eyes were all in the top 10 most common symptoms in each group, and fatigue (50%–79%) and bone aches (43%–66%) were both in the top three (Figure 1A). Although the types of symptoms were generally similar across groups, the proportions of patients reporting each symptom were often highest in the USA group. Bone aches, joint stiffness, and insomnia were reported by proportionally more patients (i.e., ≥10%) in the USA group than in both the HI or LMI groups, while a higher proportion of patients in the USA group reported headaches versus the HI group and fatigue and low mood versus the LMI group (Figure 1A).

Similarly, nine of the ten most frequently self‐reported complications were the same in each group (blood clots, fever, gallbladder stones, infections, joint issues, required blood transfusions, swollen/painful fingers or toes, tight chest pain, and vision problems). Joint issues (44%–67%) and fever (58%–69%) were both in the top three most‐common complications in each group (Figure 1B). Several complications were reported by a greater proportion of patients in the USA than the HI and LMI groups (Figure 1B). Fever, however, was reported by a lower proportion of patients in the USA than the LMI group (Figure 1B).

### 3.3 VOC burden and management

**FIGURE 2:**
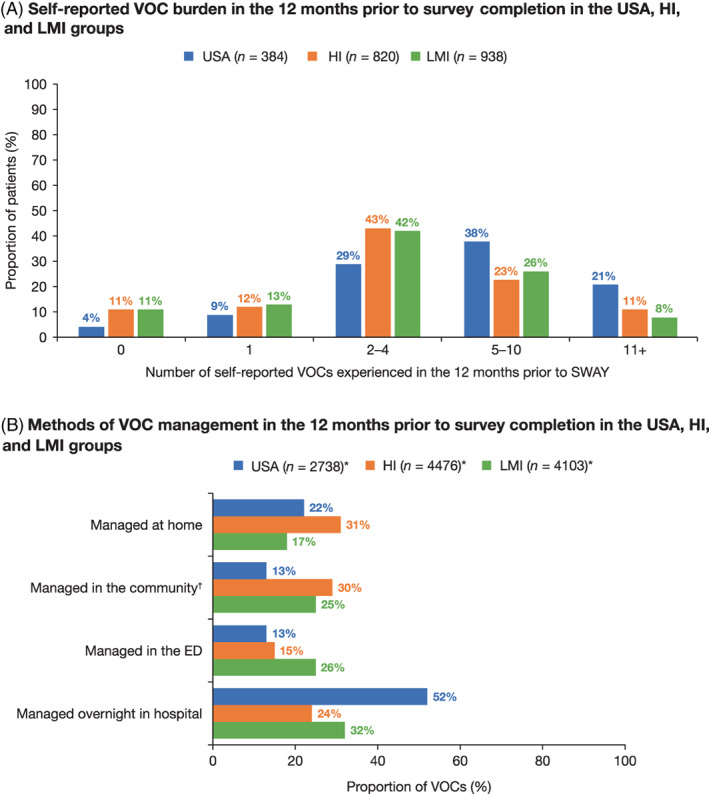
(A) Self‐reported VOC burden and (B) methods of VOC management in the 12 months prior to survey completion in the USA, HI, and LMI groups. *Sample sizes are for the total number of VOCs, rather than the number of patients. ^†^Required an HCP visit but not a visit to the ED/hospital. ED, emergency department; HCP, healthcare professional; HI, high‐income; LMI, low‐ to middle‐income; SWAY, Sickle Cell World Assessment Survey; VOC, vaso‐occlusive crisis [Color figure can be viewed at wileyonlinelibrary.com]

The self‐reported VOC burden was higher in the USA group than in the HI and LMI groups; the mean [standard deviation (SD)] number of VOCs in the 12 months prior to survey completion was 7.1 (5.7) versus 5.5 (8.9) and 4.4 (4.6), respectively. During the same period, the mean (SD) number of VOCs reported in pediatric patients (≤18 years) was 4.3 (3.0) versus 5.5 (8.9) and 4.4 (4.5) in the USA, HI, and LMI groups, respectively. In adult patients (>18 years), the mean [SD] number of VOCs was higher in the USA (7.9 [6.0]) than the HI (5.4 [8.9]) and LMI (4.3 [4.6]) groups. Corresponding median values for VOC burden can be found in Table S1. Although the proportion of patients who reported experiencing ≥1 VOC in the 12 months prior to survey completion was comparable across groups (USA, 96%; HI, 89%; LMI, 89%), the proportion experiencing ≥5 VOCs was higher in the USA group (59%) than in both the HI (34%) and LMI (34%) groups (Figure 2A).

The most frequently reported management of VOCs was overnight in hospital for both the USA and LMI groups (52% and 32%, respectively), whereas VOCs were most often managed at home in the HI group (31%). The proportion of VOCs managed in the community (i.e., requiring an HCP visit, but not a visit to the ED/hospital) was lower in the USA group than in the HI group, while the proportion managed overnight in hospital was higher in the USA group than in either the HI or LMI groups. Compared with the LMI group, the proportion of both VOCs managed in the community or in the ED was lower in the USA group (Figure 2B).

The most common reasons for managing VOCs at home were consistent across the USA, HI, and LMI groups, with poor prior experience at the ED/hospital (50%, 44%, and 23%, respectively) and the belief that medical assistance was not required (26%, 35%, and 25%, respectively) being two of the top three reasons in each group. The most common methods for managing VOCs at home were resting and/or sleeping (USA, 84%; HI, 83%; LMI, 53%) and drinking plenty of fluids (USA, 84%; HI, 86%; LMI, 51%). Taking opioid painkillers was reported by 73% and 66% of patients in the USA and HI groups, respectively, compared with only 34% in the LMI group.

### 3.4 Treatment utilization

**FIGURE 3:**
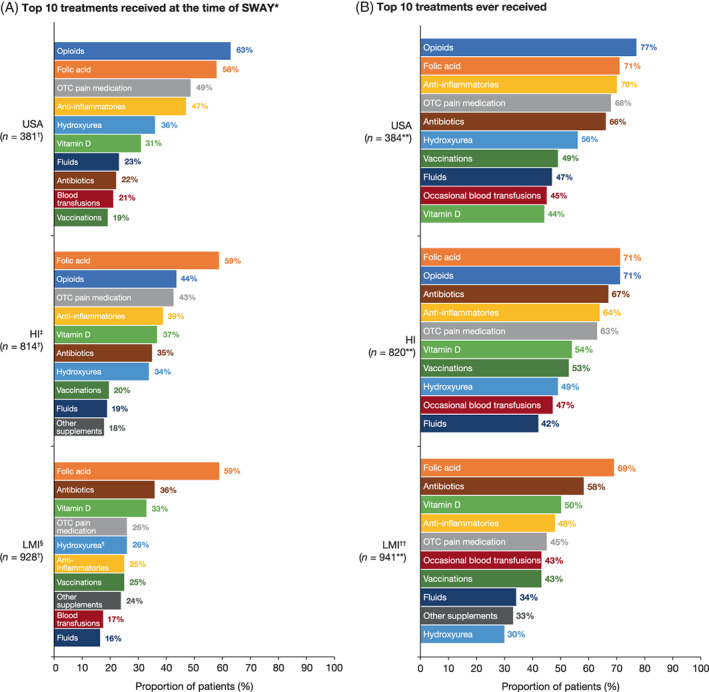
Top 10 treatments that patients in the USA, HI, and LMI groups reported (A) receiving at the time of SWAY* and (B) having ever received. *Only patients who had ever received treatment(s) for their sickle cell disease were included in this analysis (*n* = 2123). ^†^This included patients who selected ‘No current treatment’ (USA, *n* = 1; HI, *n* = 36; and LMI, *n* = 91). ^‡^Occasional blood transfusions, reported by 15% of patients, were outside of the top 10 treatments for the HI group. ^§^Opioids, reported by 13% of patients, was outside of the top 10 treatments for the LMI group. ^¶^Hydroxyurea was not available in Ghana or Nigeria at the time of SWAY. **This included patients who selected ‘No treatment ever prescribed’ (USA, *n* = 3; HI, *n* = 6; and LMI, *n* = 13). ^††^Opioids, reported by 27% of patients, was outside of the top 10 treatments for the LMI group. HI, high‐income; LMI, low‐ to middle‐income; OTC, over‐the‐counter; SWAY, Sickle Cell World Assessment Survey [Color figure can be viewed at wileyonlinelibrary.com]

At the time of the survey, a higher proportion from the USA group than both the HI and LMI groups reported current opioid use (63% vs. 44% and 13%; Figure 3A), while a lower proportion reported current antibiotic use (22% vs. 35% and 36%). In addition, a higher proportion of patients from the USA group than the LMI group reported use of over‐the‐counter (OTC) pain medication (49% vs. 26%), anti‐inflammatories (47% vs. 25%), and HU (36% vs. 26%) (Figure 3A).

The proportion of patients who had ever received specific treatments to manage their SCD was similar in the USA and HI groups except for vitamin D use (44% vs. 54%, respectively) (Figure 3B). In contrast, a higher proportion of patients in the USA than in the LMI group reported having ever received opioids (77% vs. 27%), anti‐inflammatories (70% vs. 48%), OTC pain medication (68% vs. 45%), HU (56% vs. 30%), and fluids (47% vs. 34%) (Figure 3B).

### 3.5 Treatment satisfaction

Treatment satisfaction levels were similar across the three groups, although a higher proportion of patients in the USA group than the HI group expressed strong agreement with the contrasting statements “*I would recommend my SCD treatment to another SCD patient*” (66% vs. 53%) and “*I do not want to take my SCD treatment for the foreseeable future*” (48% vs. 37%; Figure S2). While most patients in each group reported that they were very satisfied with their current treatment's control of their SCD (63%–72%), most patients also indicated a strong desire for an alternative to their current pain‐management medication (70%–76%; Figure S2 and Table S2) and concern about the long‐term side effects of their treatment (56%–66%; Figure S2). See Appendix S1 for treatment satisfaction data by prior VOCs (Figure S3).

To independently assess patient satisfaction with two key treatments—opioids, which treat a symptom of SCD, and HU, which is disease modifying—responses were also analyzed in patients who were receiving opioids but not HU at the time of SWAY, and patients who were receiving HU but not opioids. In the HI group, the proportion of patients who expressed overall satisfaction with an opioid‐based regimen's control of their SCD symptoms was lower than that of patients receiving a HU‐based disease modifying regimen (51% vs. 74%, respectively; Figure S4). This was not true for the USA group, for which patients reported similar levels of treatment satisfaction regardless of treatment type. Due to the limited availability of HU and opioids in many LMI countries, any comparisons with HI countries should be interpreted with caution.

### 3.6 Employment history and impact of SCD on employment

Of patients eligible to complete this part of the survey, the proportions in employment (either full‐time or part‐time) were similar in the USA (25%; *n* = 83/335) and LMI (19%; *n* = 125/664) groups, but higher in the HI group (50%; *n* = 362/729). A higher proportion of patients from the USA group reported not working, being on disability, or being on long‐term sick leave (61% combined; *n* = 204/335) than in the HI (16% combined; *n* = 120/729) and LMI (17% combined; *n* = 115/664) groups. For seven of the nine statements, a higher proportion of patients from the USA group than both the HI and LMI groups reported that SCD had had a negative impact on work life (Figure S5A).

A higher proportion of patients in the USA group strongly agreed that SCD had prevented them from attending work, impaired their ability to keep a job, prevented them from finding a suitable job, and caused them to be turned down at job interviews compared with the HI and LMI groups; prevented them from being promoted at work compared with the HI group only; and limited them to certain careers, and prevented them from progressing further in their career compared with the LMI group only (Figure S5B).

See Appendix S1 for data on the impact of SCD on schooling (Figure S6).

## 4 DISCUSSION

This analysis of SWAY demonstrated that patients with SCD from the USA reported a higher symptom/complication burden than both the HI and LMI groups. The only symptom/complication that was reported less often (i.e., differed by ≥10%) by USA patients was fever (vs. LMI patients only). This may be related to public health measures in the USA that have helped to reduce infection, such as penicillin prophylaxis and nationwide vaccination programs. However, it is likely that the higher symptom/complication burden is not reflective of more severe disease in the USA than in HI and LMI countries, but instead relates to various factors. For example, availability and access to healthcare are influenced, in part, by the wealth of a country and the relative allocation of resources to healthcare. Therefore, it is interesting to note that according to the World Bank, the gross domestic product (GDP) for the USA is approximately 15 times greater than the average GDP of the other HI countries in SWAY (USA GDP [2020]: US$20 937 billion; average HI GDP [2019/20]: US$1443 billion).^15^ Other factors contributing to the differences between the USA and HI group include: the structure of the healthcare systems and associated access to healthcare, with insurance‐based healthcare in the USA focusing on complications; differences in the climate; and cultural differences. The factors contributing to the differences between the USA and LMI group are likely to include: early diagnosis in the USA due to the availability of screening programs; differences in the pediatric‐to‐adult transition of care and in access to adult care; proactive patient engagement with comprehensive care that focuses on complications; the availability of diagnostic approaches to assess and record disease burden; a higher average age in the USA group; differences in climate; and the availability of, and access to, healthcare services.

There are likely several reasons for the reported higher VOC burden in the USA group than in both the HI and LMI groups. The US campaign to identify pain as the “fifth vital sign”^16^ may have increased patients' willingness to report pain and/or the pressure on physicians to address patients' pain compared with other HI and LMI countries. Other possible reasons include survival bias (i.e., patients in the USA who survive infection and live long enough to experience more pain) and climate conditions (some parts of the USA experience substantially colder weather than other countries that participated in SWAY, particularly India and those in the Middle East and Africa).

Not only was the VOC burden in the USA group higher than in HI and LMI countries (mean 7.1 vs. 5.5 and 4.4, respectively, in the 12 months prior to SWAY), it was substantially higher than reported in previous studies.^17^ However, the reported VOC rates in these other studies are likely to be an underestimation as home‐managed VOCs are rarely included in such analyses. For example, 23% of the 31 017 patient‐days analyzed in one study were due to VOCs that did not require healthcare utilization (i.e., home‐managed VOCs).^17^

The proportion of VOCs that patients reported as requiring overnight hospital stays in the USA group was approximately twice that of both the HI and LMI groups (52% vs. 24% and 32%, respectively). Various factors could contribute to the reliance on hospital treatment in the USA, including a healthcare system that is heavily centered around ED/hospital care, combined with insurance‐based healthcare and the existing liability culture, as well as easy access to hospital care and the potential lack of providers who feel comfortable treating pain in the community. The dependence on hospitals for managing VOCs suggests that SCD carries a higher societal burden and has a greater impact on healthcare resource utilization in the USA than in other HI and LMI countries. Around one‐fifth (22%) of VOCs experienced in the USA were managed at home according to patient reports, which is a lower rate than observed in other studies. In the PiSCES study (*n* = 232; aged 16–64 years), 10.6%–19.8% of reported crises were cared for in the hospital setting, implying that a large proportion were managed at home (although this analysis did not consider community‐based management of VOCs).^18^ In a more recent US survey of 303 adults with SCD, 51% of participants who experienced ≥1 VOC in the 12 months preceding survey completion reported that they managed their VOCs at home.^19^ In SWAY, the most common reason for managing VOCs at home was because of poor prior experience at the ED/hospital, which is perhaps not surprising given the stigma associated with seeking pain relief in the ED.^20^

Reported current use of opioids was particularly high among patients in the USA group (63%) compared with the HI (44%) and LMI (13%) groups. This finding was reflected in two recent studies: one that reported that 240 million opioid prescriptions were dispensed in the USA in 2015 (equivalent to almost one prescription for every adult in the general population)^21^ and another SCD‐based study in the USA that identified that each VOC was associated with an increased use of opioids.^22^ The US campaign to identify pain as the fifth vital sign has been a key driver in compelling providers to prescribe opioids for pain.^16^ It is important to note that while opioids (both intravenous and oral) remain a valid treatment option for patients with SCD,^23^ their availability is country dependent, and particularly limited in many countries across Africa and the Middle East.

Overall levels of treatment satisfaction were similar between the USA group and the HI and LMI groups. It seems contradictory that patients in the USA, who reported a high VOC burden, also express a comparatively high level of satisfaction with their treatment's control of their SCD. This paradox is also reflected in patients who received opioid‐based and HU‐based regimens (Figure S4). Most patients, regardless of geographical location, also expressed a strong desire for alternative pain medication options and concern over the long‐term effects of their treatment. This highlights the global unmet need for new SCD treatments that provide better disease control and greater pain relief, with fewer long‐term effects and reduced stigma.

A consistently higher proportion of patients from the USA than from other HI and LMI countries reported a high impact of SCD on their education (see Appendix S1), which is aligned with previous studies reporting the negative effect of SCD on both school attendance^24^ and performance^25^ in the USA. This could be related to differences in schooling systems and access to education. Effective and meaningful training of educators on the unpredictable nature and impact of SCD on health and daily life remain a key unmet global need.

Similarly, proportionally, more patients from the USA than from other participating countries in SWAY reported a high impact of SCD on various aspects of their work life and career progression, supporting multiple USA‐based studies that noted the substantial negative impact of SCD on employment.^26^, ^27^, ^28^ The USA group had a higher proportion of patients not working, on disability, or on sick leave than the other HI and LMI countries. Notably, more patients from the USA group had been dismissed from their job due to circumstances related to SCD than in the HI and LMI groups (54% vs. 27% and 23%, respectively). This could be accounted for by various factors not explored in this study. However, these data are highly indicative of the stigma that surrounds SCD and highlight the urgent need for increased awareness and deeper understanding about SCD from employers around how they can optimally support their employees with SCD.

### 4.1 Limitations

A key strength of SWAY is that it captures real‐world data with a global reach. Nevertheless, the study does have limitations, which are described in full in the primary analysis (including discussion of the lower mean age and higher proportion of proxy completers in the LMI group than in the USA group).^8^ One key limitation of the current analysis is the classification of countries as either HI or LMI. While this broadly allows the identification of differences in the data based on a country's economic status, it does not fully address differences in culture (e.g., willingness to disclose personal disease‐related patient information, social attitude, and stigma towards SCD), healthcare resource availability, patient engagement, and education among HCPs, factors that may also differentially affect the experiences of patients from different countries within a group. Furthermore, we recognize the selection bias in the enrollment methods that may affect the interpretation of these data; participants may not be fully representative of that country's overall SCD population. However, the unique nature of this study in attempting to capture attitudes and experiences of patients with SCD across the globe still produces meaningful data. It is important to note that a higher level of reporting does not necessarily reflect a higher burden of disease, since recall bias and a variety of socioeconomic and cultural factors likely affect the willingness of patients to report personal disease‐related information. The differing availability of some treatments (notably HU and opioids) in participating countries also limits the ability of patients to comment on their use and treatment satisfaction. Finally, patient sampling in many SWAY‐participating countries was limited compared with the USA, so may be less representative of the country's SCD population.

## 5 CONCLUSIONS

Data presented here further support our previous finding that the burden of SCD on patients' lives is substantial regardless of geographical location.^8^ However, by focusing on the USA patient population, we note that there is a higher patient‐reported disease burden and healthcare resource utilization in the USA than in other participating countries. While this is unlikely to reflect a more severe disease burden attributable to geographical location, it does support the need for a substantial improvement in disease awareness and understanding of SCD. Patients in the USA expressed a high level of satisfaction with their treatment's control of their SCD, despite having a high VOC burden. Pain is a defining symptom of SCD. This is reflected by the consistently high desire for alternative pain management treatments, irrespective of geographical location, emphasizing the global unmet need for new treatments to reduce the long‐term impact of vaso‐occlusion. New treatments, including those that reduce pain but also decrease the demand on healthcare resources, are urgently needed both within the USA and globally.

## AUTHOR CONTRIBUTIONS

All authors (except Jincy Paulose, Nicholas Ramscar, Tom Bailey, and Olivera Rajkovic‐Hooley) were members of the SWAY steering committee, of which Ifeyinwa Osunkwo and John James were co‐chairs. All authors contributed to the survey design and data interpretation, and reviewed and approved the report. John James and Cassandra Trimnell contributed to patient recruitment.

## FUNDING INFORMATION

Novartis provided sponsorship and was involved in running the survey. Adelphi Real World (ARW) designed the survey in collaboration with the SWAY steering committee and Novartis. Funding was provided by Novartis Pharmaceuticals Corporation to ARW for the survey design, data collection, and data analysis, and to Mudskipper Business Ltd, Bollington, Macclesfield, UK for medical writing support.

## CONFLICT OF INTEREST

I.O. reports consultancy for Agios, Cheisi, Cyclerion, FORMA Therapeutics, Global Blood Therapeutics, HCP Live, Novartis, and Takeda; speakers' bureau for Emmaus, Global Blood Therapeutics, and Novartis; advisory board for Acceleron, Cyclerion, Cheisi, Emmaus, FORMA therapeutics, Global Blood Therapeutics, Novartis, and Novo‐Nordisk; grants from Centers for Disease Control (CDC), Department for Public Health (DPH), Health Resources and Services Administration (HRSA), Patient Centered Outcomes Research Institute (PCORI), and NC; Data and Safety Monitoring Board (DSMB) membership for Micella Biopharma; and Editor‐In‐Chief for *Hematology News*. J.J. reports employment by the Sickle Cell Society and honoraria from Novartis. F.E.‐R. reports research funding from Cyclerion, Novartis, and Pfizer; and advisory board participation for Novartis, Global Blood Therapeutics, and bluebird bio. A.N. reports consultancy/expert testimony for bluebird bio, Global Blood Therapeutics, and Novartis. C.P.M. reports consultancy for Agios, bluebird bio, Emmaus, Forma Therapeutics, Global Blood Therapeutics, Novartis, Roche, and Sanguine. C.T. reports consultancy/expert testimony for Novartis, Cyclerion, and Global Blood Therapeutics. J.P. is an employee of Novartis Pharmaceuticals Corporation. N.R. is an employee of Novartis Pharma AG. O.R.‐H. and T.B. are employees of Adelphi Real World, which received payment from Novartis Pharmaceuticals as part of this research. B.A. reports consultancy or membership on an advisory committee for Agios, Aruvant, bluebird bio, CRISPR/Vertex, Cyclerion, Emmaus, Forma Therapeutics, Global Blood Therapeutics, Hemanext, Novartis, Novo Nordisk, Sanofi Genzyme, and Terumo BCT; and research funding from Forma Therapeutics, Global Blood Therapeutics, Hemanext, Imara, and Novartis.

## Supporting information


Appendix S1

## Data Availability

All authors had access to the raw combined data, but not individual patient data to ensure patient anonymity. The decision to submit the manuscript for publication was made by all authors.
