# Supplementary material for: Burden of disease, treatment utilization, and the impact on education and employment in patients with sickle cell disease: A comparative analysis of high‐ and low‐ to middle‐income countries for the international Sickle Cell World Assessment Survey
Source: Am J Hematol. 2022 Jun 20;97(8):1055–64. doi: 10.1002/ajh.26576 (PMC9540420; doi:10.1002/ajh.26576)
Supplement: Supplementary file 1 — Appendix S1 [file AJH-97-1055-s001.pdf]

Supplementary Material to:

Burden of disease, treatment utilization, and the impact on education and employment in patients with sickle cell disease: A comparative analysis of high- and low- to middle-income countries for the international Sickle Cell World Assessment Survey (SWAY)

Ifeyinwa Osunkwo, John James, Fuad El-Rassi, Alecia Nero, Caterina P. Minniti, Cassandra Trimnell, Jincy Paulose, Nicholas Ramscar, Tom Bailey, Olivera Rajkovic-Hooley and Biree Andemariam

Table S1. Patient-reported median number of VOCs in the 12 months prior to survey completion for the total population, and by patient age

	USA	HI	LMI
**Total population, *n***	384	820	940
Median (IQR) number of VOCs	6.0 (3.0-10.0)	3.0 (2.0-6.0)	3.0 (2.0-6.0)
**Pediatric patients, aged ≤18 years, *n***	82	222	523
Median (IQR) number of VOCs	4.0 (1.8-6.2)	3.0 (1.0-6.0)	3.0 (2.0-6.0)
**Adult patients, aged >18 years, *n***	302	598	417
Median (IQR) number of VOCs	6.0 (3.0-11.2)	3.0 (2.0-6.0)	3.0 (2.0-5.0)

Abbreviations: HI, high-income; IQR, interquartile range; LMI, low- to middle-income; VOC, vaso-occlusive crisis

Table S2. Paired responses of strong agreement[a] with treatment satisfaction statements of patients in the USA group

	Treatment satisfaction statement	*n* (*N*)	%
**Patients who expressed a strong agreement[a] with the statement…**	***…"Overall, I am very satisfied with my treatment's control of my SCD"…***	**274 (375)**	**73**
**…also expressed strong agreement[a] with the statements…**	…"*I would recommend my SCD treatment to another SCD patient*"	210 (274)	77
	…"*I do not wish to take my SCD treatment for the foreseeable future*"	149 (274)	54
	…'*I wish there was an alternative treatment to my current pain management medication*"	186 (237)[b]	78
**Patients who expressed a strong agreement[a] with the statement…**	***…"My treatment is effective at treating my SCD symptoms"…***	**275 (370)**	**74**
**…also expressed high agreement[a] with the statements…**	…"*I would recommend my SCD treatment to another SCD patient*"	214 (275)	78
	…"*I do not wish to take my SCD treatment for the foreseeable future*"	149 (275)	54
	…"*I wish there was an alternative treatment to my current pain management medication*"	186 (236)[b]	79

Abbreviations: SCD, sickle cell disease; SWAY, Sickle Cell World Assessment Survey

[a]Patients who responded with a score of 5-7 on the corresponding 7-point Likert scale

[b]Only patients who were receiving pain medication at the time of SWAY responded to this statement

Figure S1. Countries participating in SWAY

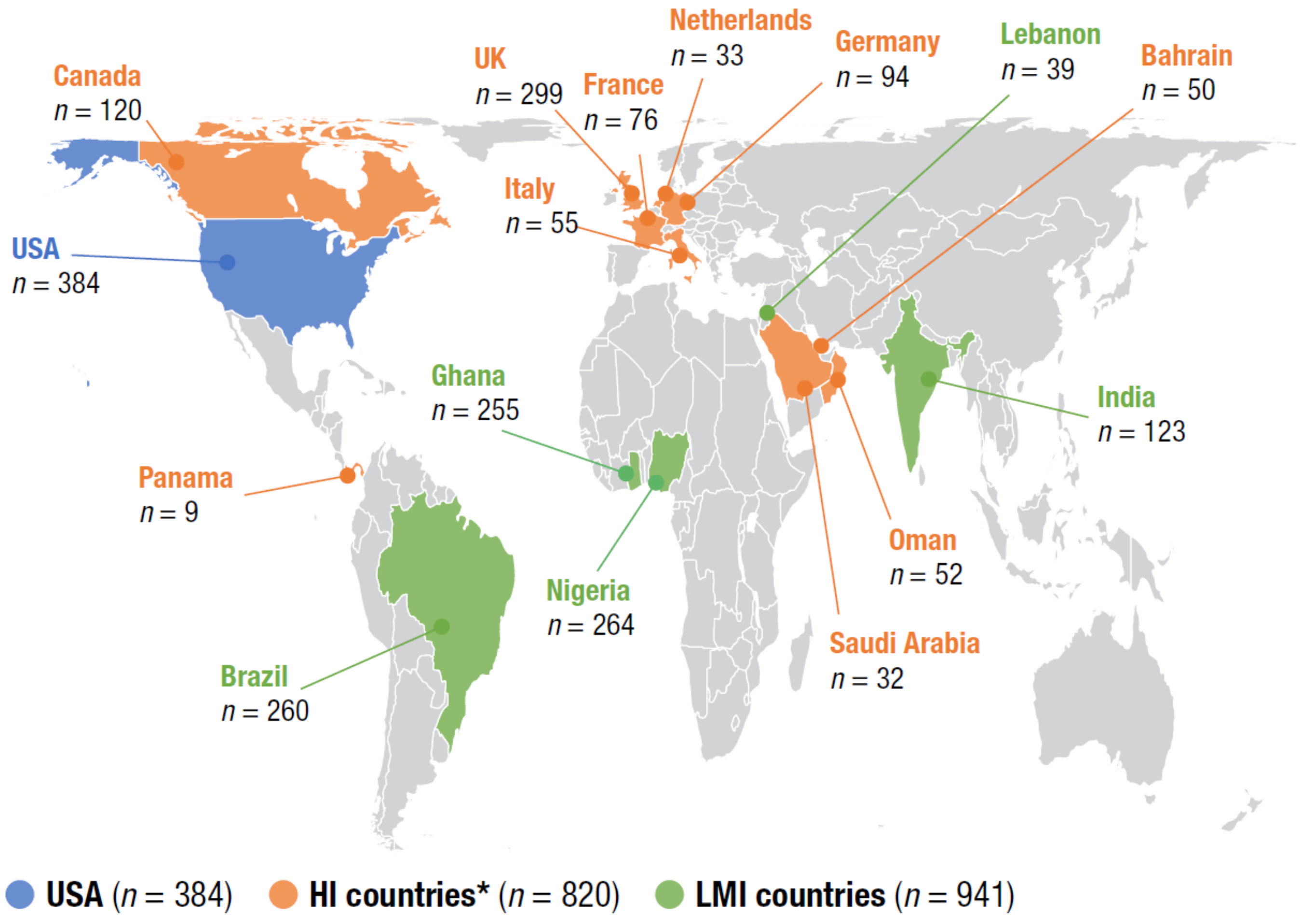

*Excluding USA. HI, high-income; LMI, low- to middle-income; SWAY, Sickle Cell World Assessment Survey

Treatment satisfaction

Figure S2. Patients' satisfaction* with treatments received at time of SWAY

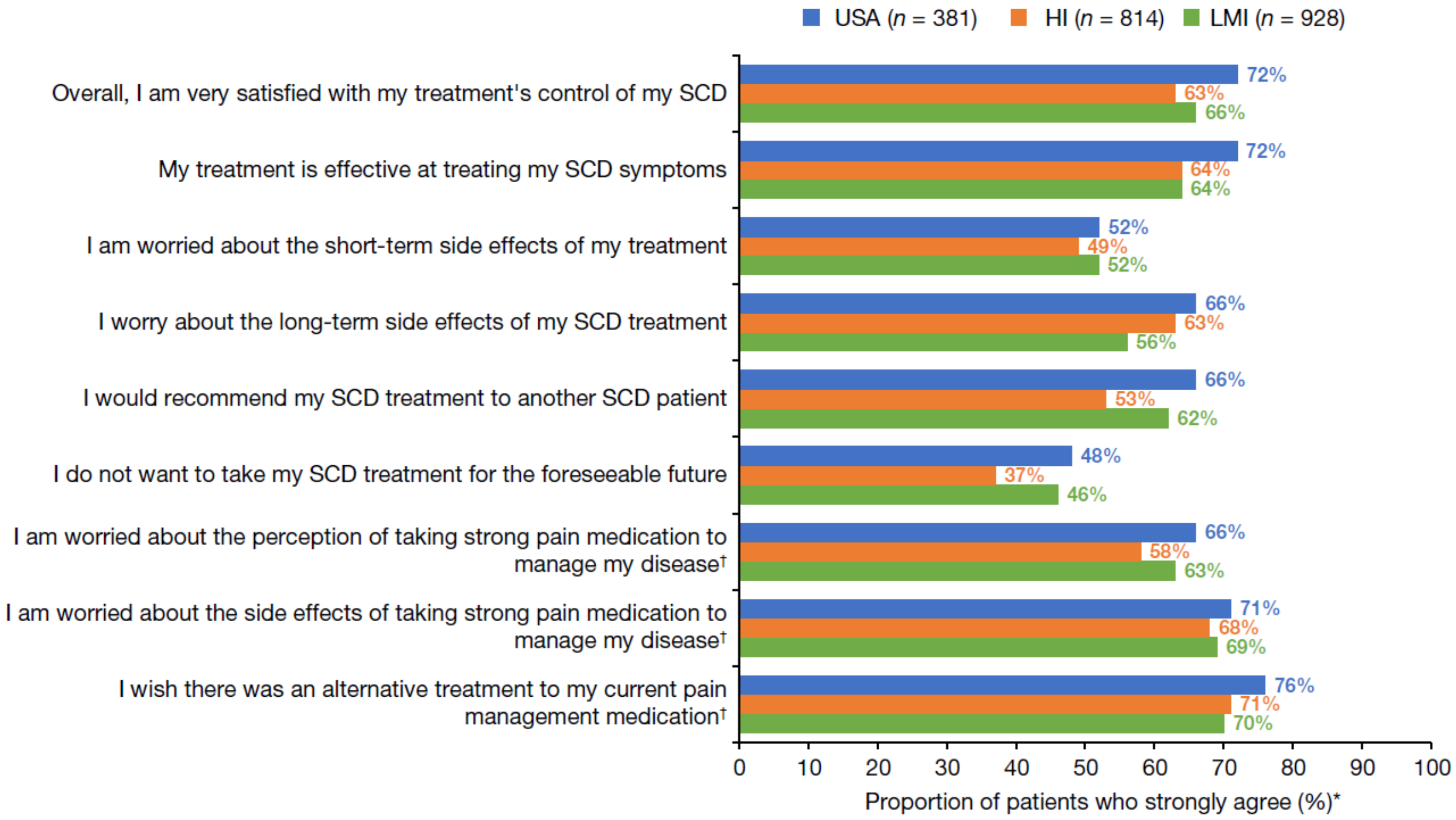

*Patients who responded with a score of 5-7 on the corresponding 7-point Likert scale. †For USA, *n* = 327; for HI, *n* = 607; and for LMI, *n* = 419. HI, high-income; LMI, low- to middle-income; SCD, sickle cell disease; SWAY, Sickle Cell World Assessment Survey

With few exceptions, treatment satisfaction levels in patients who reported 0-4 vaso-occlusive crises (VOCs) in the year prior to survey completion were comparable across all three groups (**Figure S3A**). While this trend was also true for patients from the USA and low- to middle-income (LMI) groups who reported ≥5 VOCs, a higher proportion of patients from the USA group than the high-income (HI) group strongly agreed with: "*Overall, I am satisfied with my treatment's control of my SCD*"; "*My treatment is effective at treating my SCD symptoms*"; "*I would recommend my SCD treatment to another SCD patient*"; and "*I do not want to take my SCD treatment for the foreseeable future*" (**Figure S3B**).

Figure S3. Patients' satisfaction* with treatments reported by patients who reported (A) 0-4 VOCs and (B) ≥5 VOCs in the 12 months preceding SWAY

(A) 0-4 VOCs

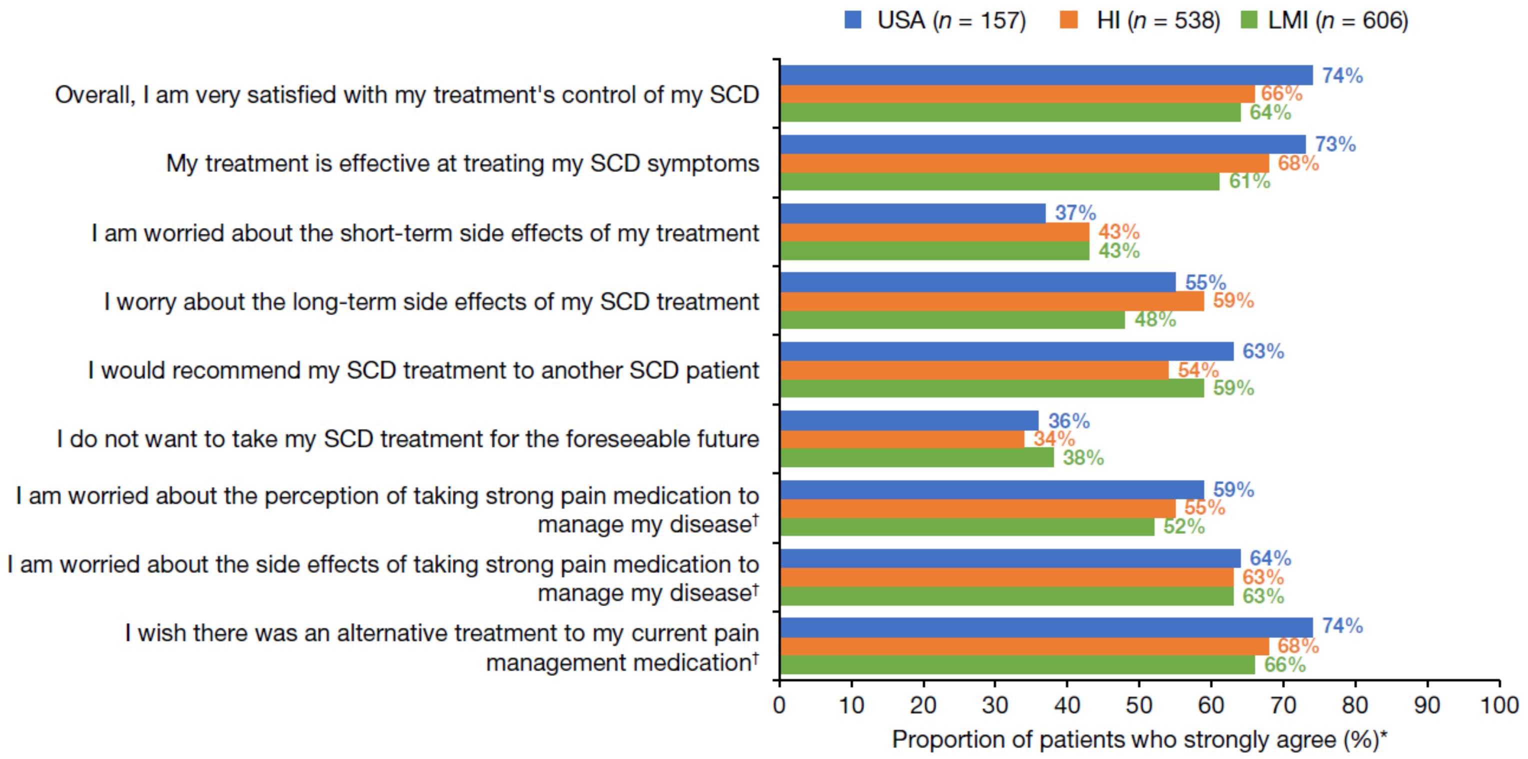

(B) ≥5 VOCs

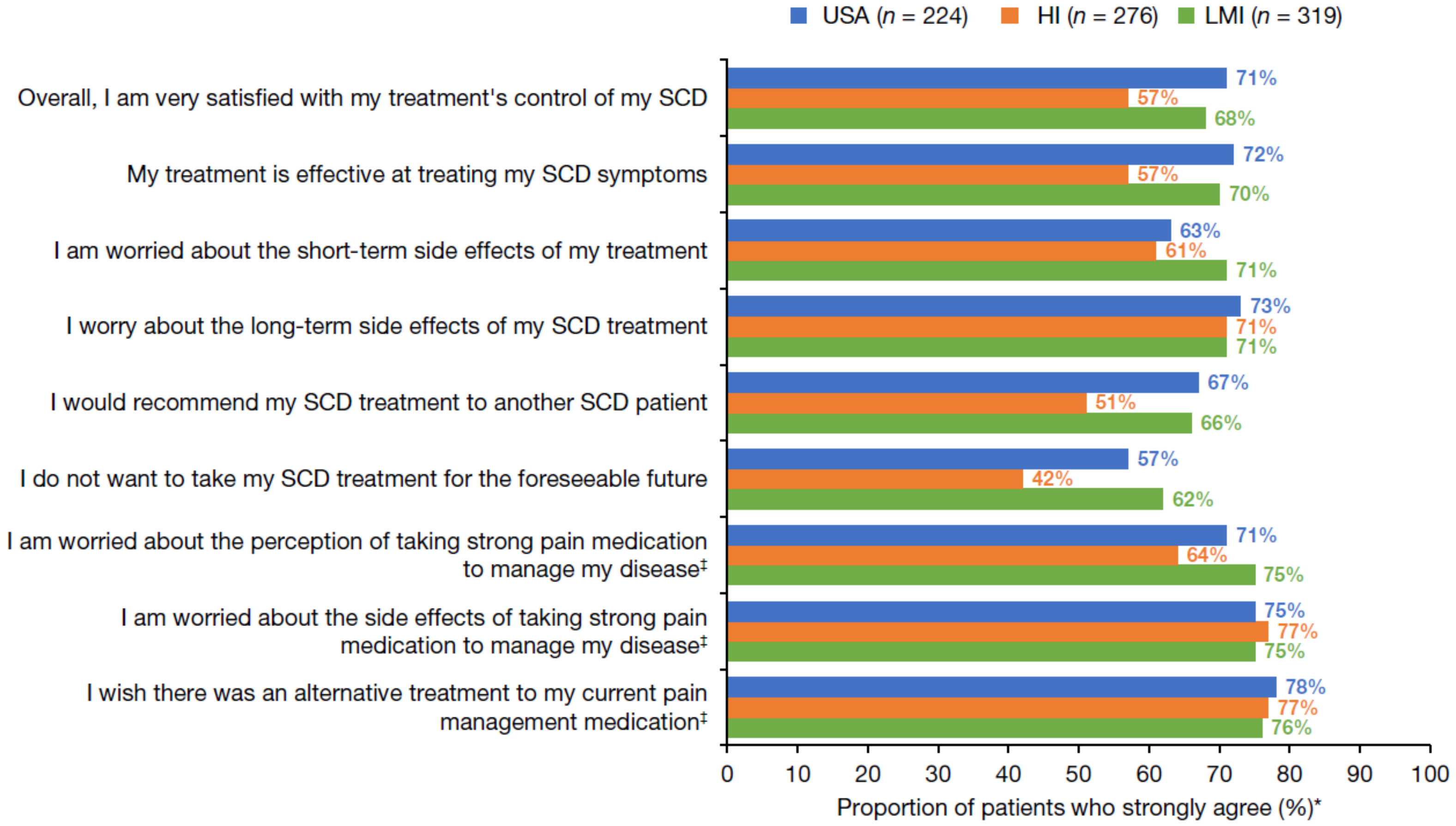

*Patients who responded with a score of 5–7 on the corresponding 7-point Likert scale. †For USA, *n* = 129; for HI, *n* = 385; and for LMI, *n* = 225. ‡For USA, *n* = 198; for HI, *n* = 222; and for LMI, *n* = 191. HI, high-income; LMI, low- to middle-income; SCD, sickle cell disease; SWAY, Sickle Cell World Assessment Survey; VOC, vaso-occlusive crisis

Figure S4. Patients' satisfaction* with (A) opioid-based and (B) HU-based treatment regimens received at the time of SWAY

(A) Opioid-based regimens†

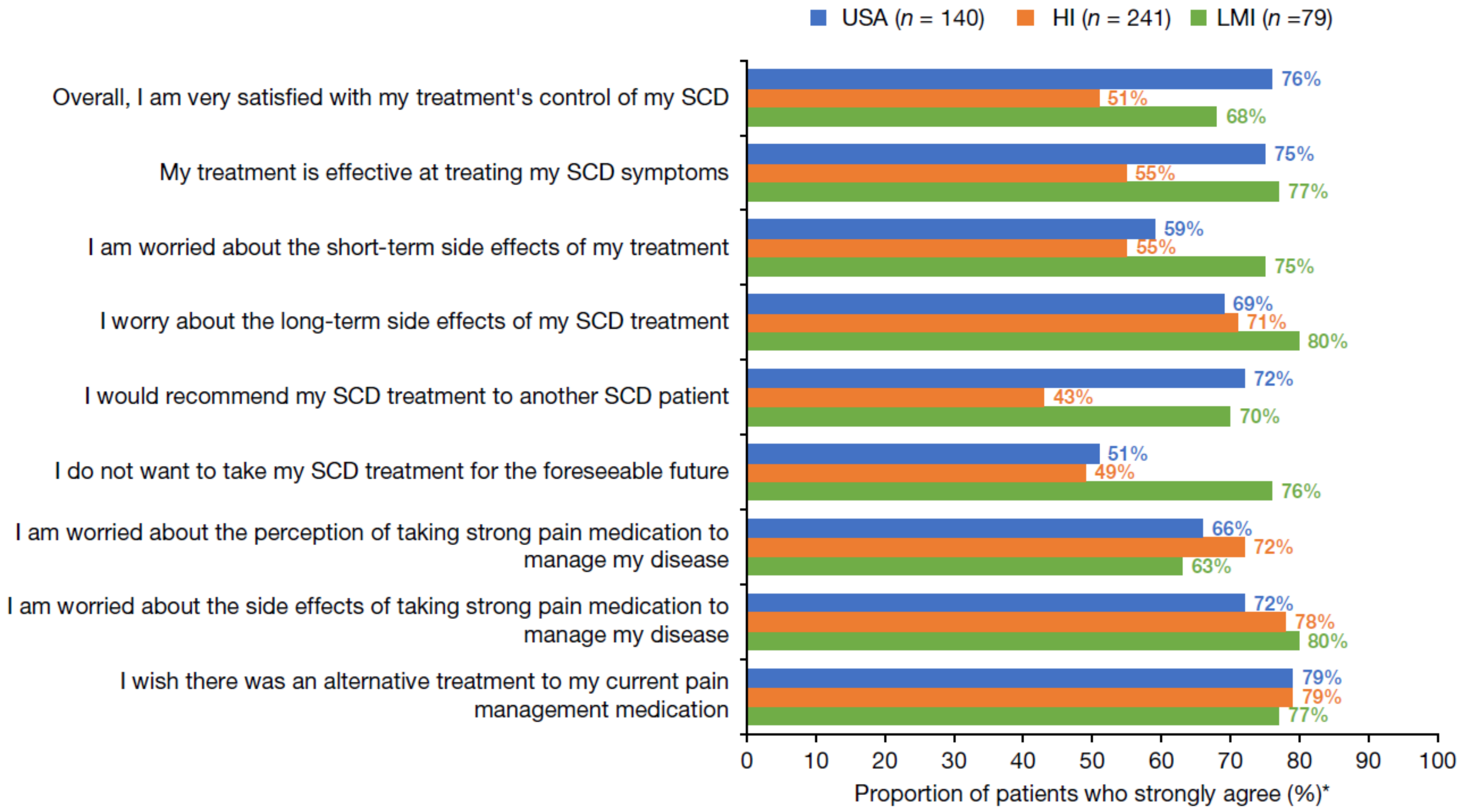

(B) HU-based regimens†

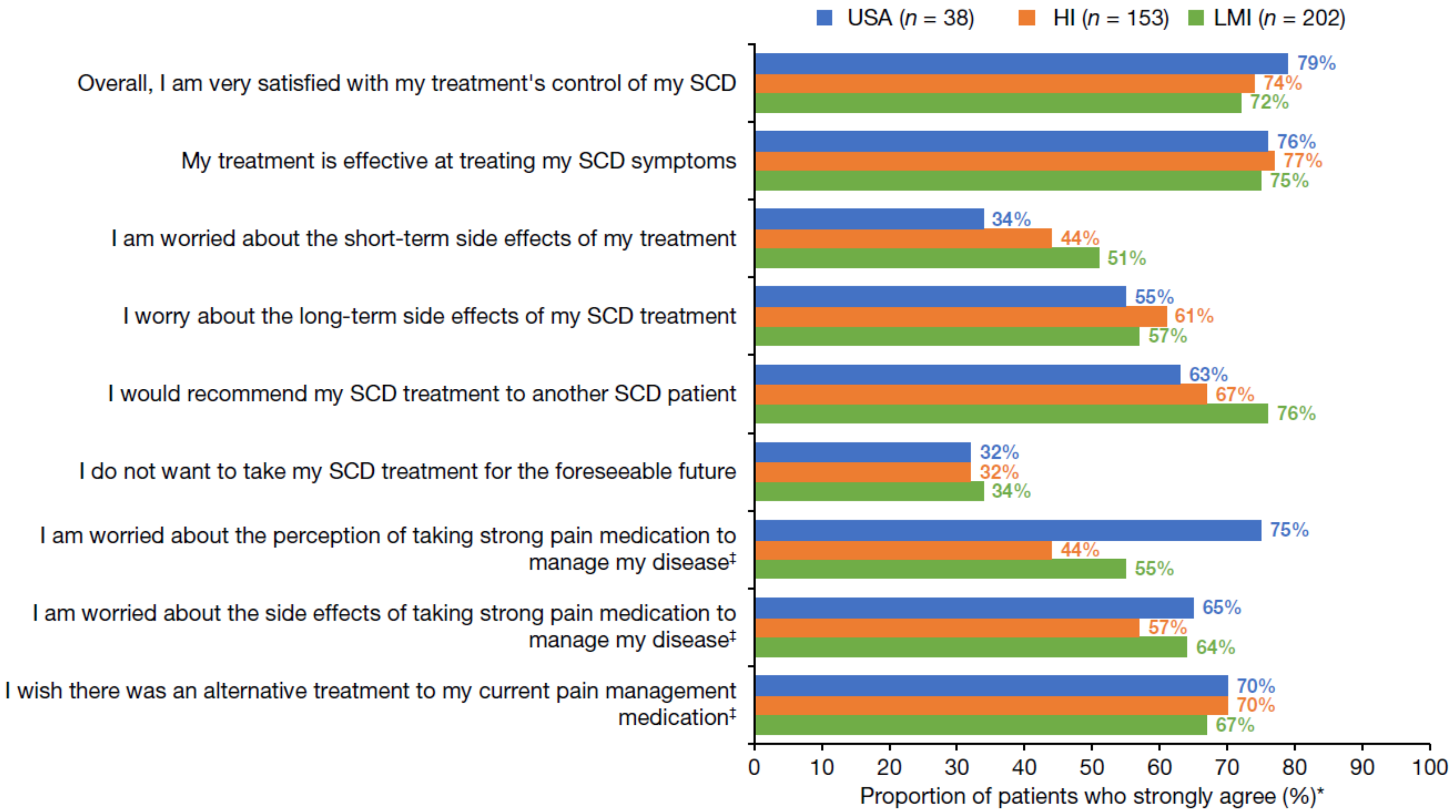

*Patients who responded with a score of 5-7 on the corresponding 7-point Likert scale. †Opioid-based regimens were defined as those containing opioids, but not HU. HU-based regimens were defined as those containing HU, but not opioids. ‡For USA, *n* = 20; for HI, *n* = 82; and for LMI, *n* = 107. HI, high-income; HU, hydroxyurea; LMI, low- to middle-income; SCD, sickle cell disease; SWAY, Sickle Cell World Assessment Survey

Figure S5. Impact* of SCD on (A) work life and (B) careers

(A) Work life

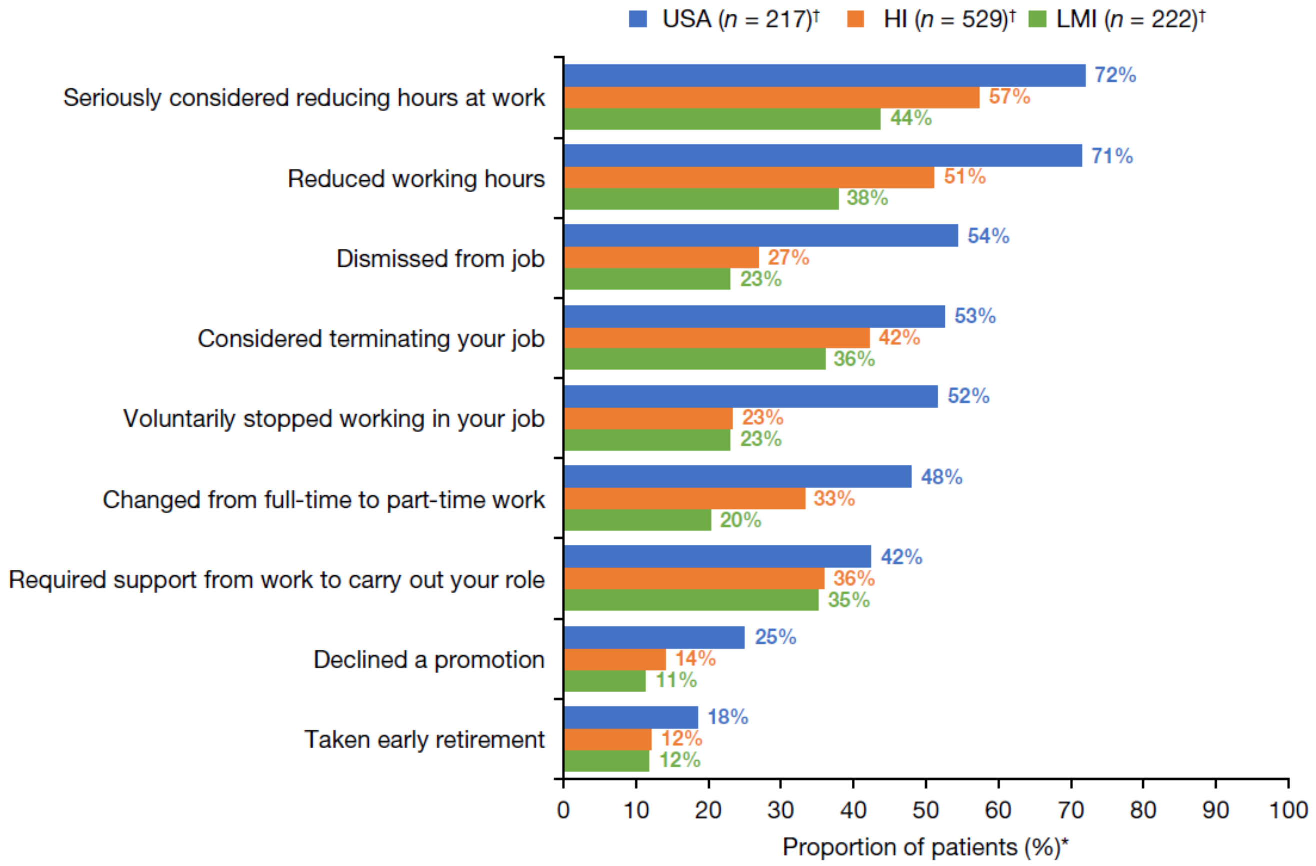

(B) Careers

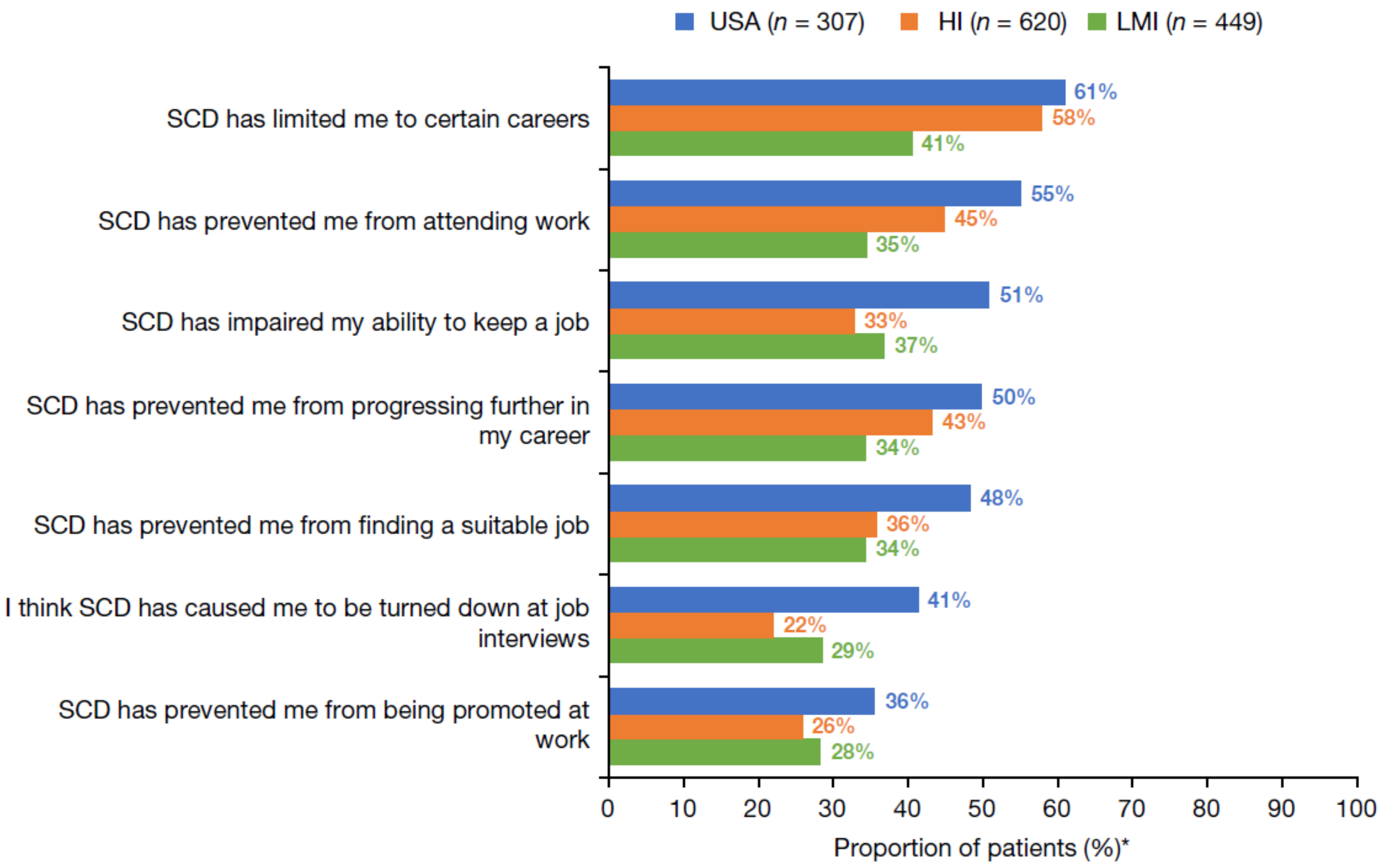

*Patients who responded with a score of 5-7 on the corresponding 7-point Likert scale. †Sample sizes include only patients who were currently employed or had history of employment. HI, high-income; LMI, low- to middle-income; SCD, sickle cell disease

Impact of SCD on schooling

A higher proportion of patients aged ≥18 years from the USA than the HI and LMI groups strongly agreed with all statements relating to the impact that SCD had on their schooling (**Figure S6A**). For patients aged <18 years, a higher proportion from the USA group than the HI group strongly agreed with all statements regarding the ongoing impact of SCD on their schooling (**Figure S6B**). In contrast, the proportions of patients who reported strong agreement were comparable between the USA and LMI groups for most statements (**Figure S6B**). The two exceptions were for "*SCD has had a bad effect on my performance in school tests*" (51% vs 37%, respectively) and "*SCD has caused me to repeat a year or retake a class at school*" (42% vs 32%, respectively; **Figure S6B**).

Figure S6. Impact* of SCD on (A) education for patients aged ≥18 years and (B) ongoing education for patients aged <18 years

(A) Impact of SCD on education for patients aged ≥18 years

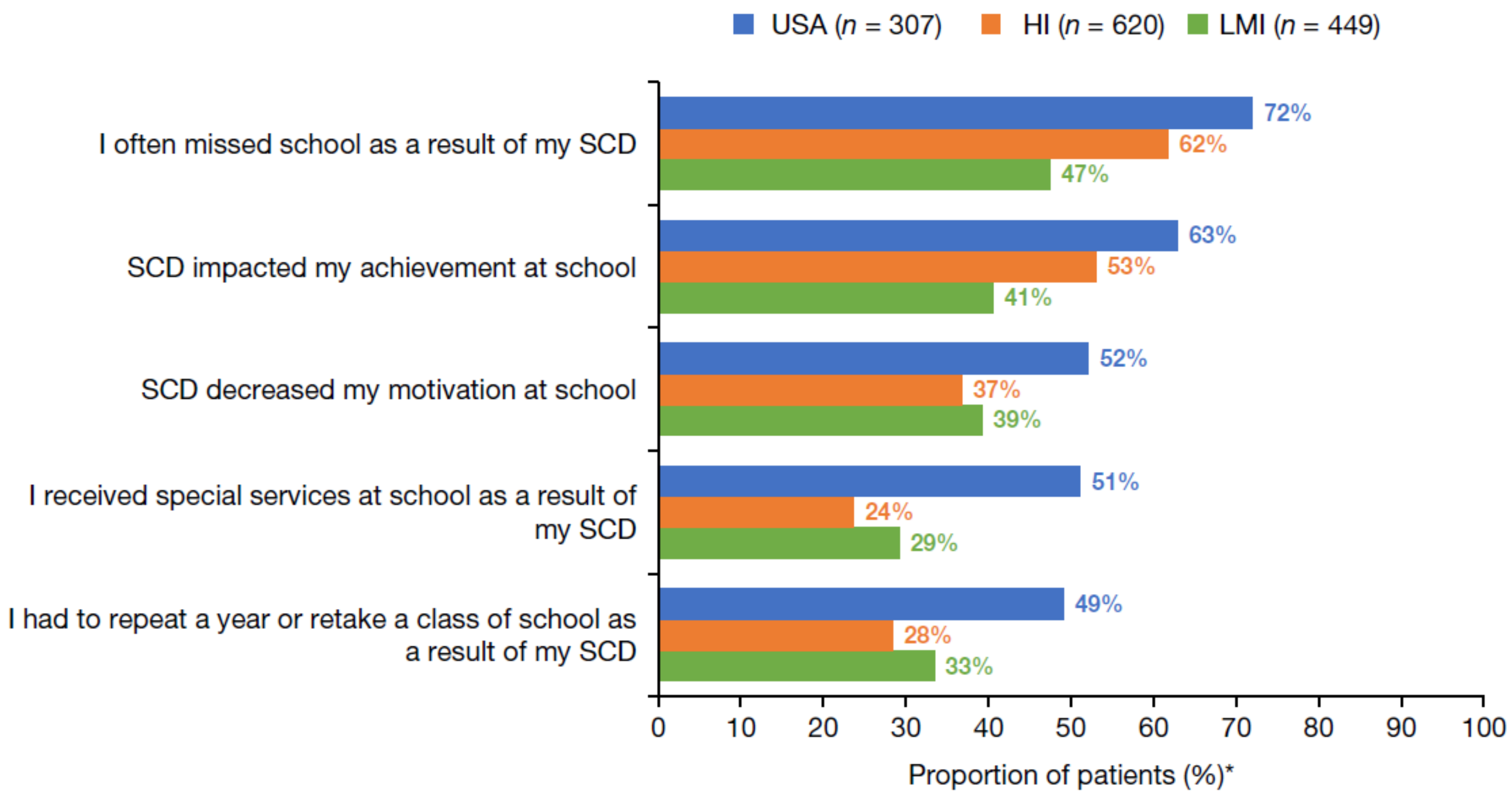

(B) Impact of SCD on ongoing education for patients aged <18 years

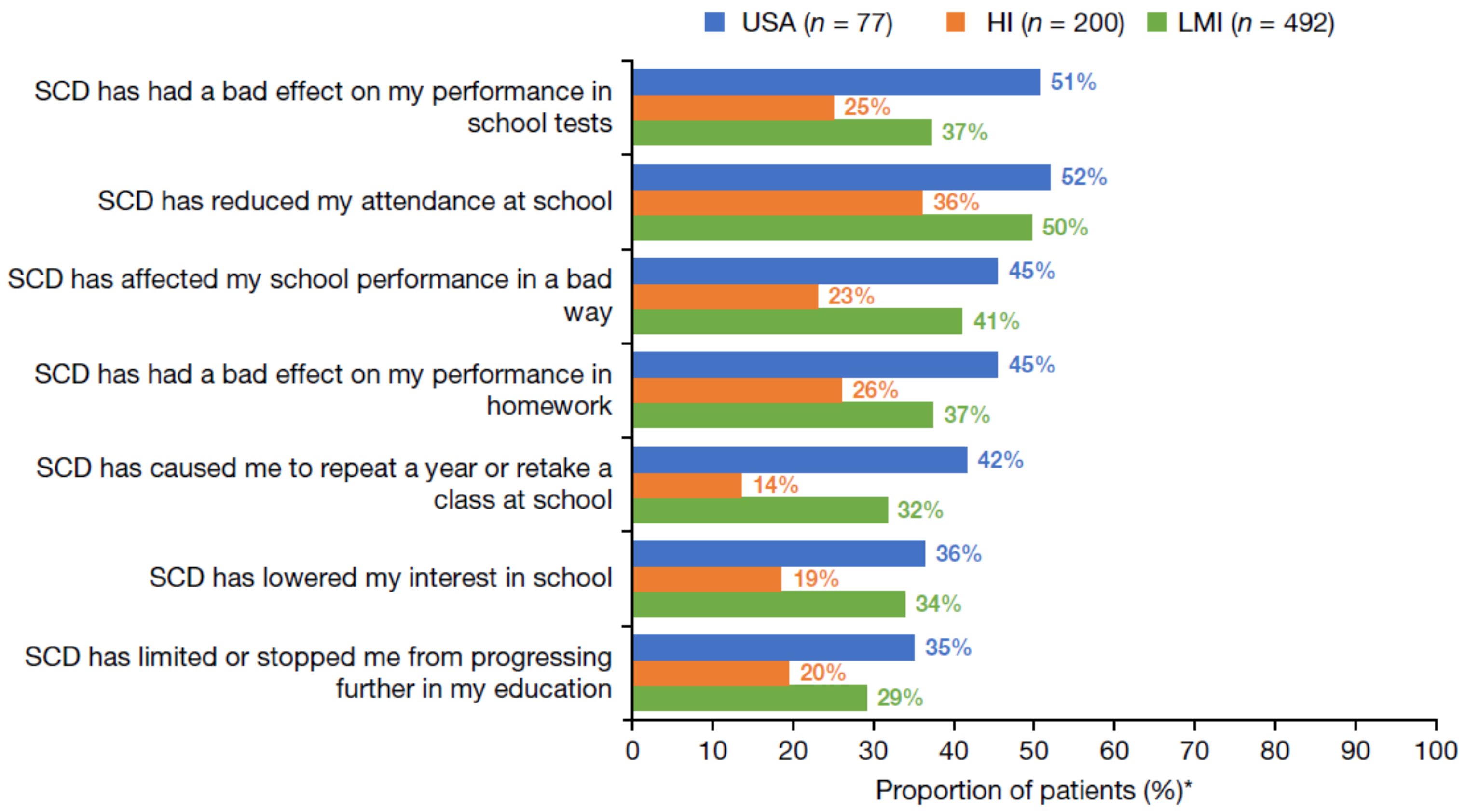

*Patients who responded with a score of 5-7 on the corresponding 7-point Likert scale. HI, high-income; LMI, low- to middle-income; SCD, sickle cell disease
